# Gut microbiome structure and function in asymptomatic diverticulosis

**DOI:** 10.1186/s13073-024-01374-9

**Published:** 2024-08-23

**Authors:** Xinwei Hua, Jessica McGoldrick, Nour Nakrour, Kyle Staller, Daniel Chulyong Chung, Ramnik Joseph Xavier, Hamed Khalili

**Affiliations:** 1 Department of Cardiology, State Key Laboratory of Vascular Homeostasis and Remodeling, and Institute of Vascular Medicine, Peking University Third Hospital, Beijing, China; 2 Division of Gastroenterology, Massachusetts General Hospital and Harvard Medical School, Boston, MA USA; 3 Clinical and Translation Epidemiology Unit, Massachusetts General Hospital and Harvard Medical School, Boston, MA USA; 4 Department of Radiology, Massachusetts General Hospital and Harvard Medical School, Boston, MA USA; 5 Broad Institute of Massachusetts Institute of Technology and Harvard, Cambridge, MA USA; 6 Institute of Environmental Medicine, Karolinska Institutet, C6 Institutet För Miljömedicin, C6, CVD-NUT-EPI Wolk, Stockholm, 171 77 Sweden; 7 Crohn’s and Colitis Center, Massachusetts General Hospital, 165 Cambridge Street, 9Th Floor, Boston, MA 02114 USA

**Keywords:** Gut microbiome, Asymptomatic diverticulosis, Metagenomic sequencing

## Abstract

**Background:**

Colonic diverticulosis, the most common lesion found in routine colonoscopy, affects more than 50% of individuals aged ≥ 60 years. Emerging evidence suggest that dysbiosis of gut microbiota may play an important role in the pathophysiology of diverticular disease. However, specific changes in microbial species and metabolic functions in asymptomatic diverticulosis remain unknown.

**Methods:**

In a cohort of US adults undergoing screening colonoscopy, we analyzed the gut microbiota using shotgun metagenomic sequencing. Demographic factors, lifestyle, and medication use were assessed using a baseline questionnaire administered prior to colonoscopy. Taxonomic structures and metabolic pathway abundances were determined using MetaPhlAn3 and HUMAnN3. We used multivariate association with linear models to identify microbial species and metabolic pathways that were significantly different between asymptomatic diverticulosis and controls, while adjusting for confounders selected *a priori* including age at colonoscopy, sex, body mass index (BMI), and dietary pattern.

**Results:**

Among 684 individuals undergoing a screening colonoscopy, 284 (42%) had diverticulosis. Gut microbiome composition explained 1.9% variation in the disease status of asymptomatic diverticulosis. We observed no significant differences in the overall diversity of gut microbiome between asymptomatic diverticulosis and controls. However, microbial species *Bifidobacterium pseudocatenulatum* and *Prevotella copri* were significantly enriched in controls (*q* value = 0.19 and 0.14, respectively), whereas *Roseburia intestinalis*, *Dorea *sp. CAG:317, and *Clostridium *sp. CAG: 299 were more abundant in those with diverticulosis (*q* values = 0.17, 0.24, and 0.10, respectively). We observed that the relationship between BMI and diverticulosis appeared to be limited to carriers of *Bifidobacterium pseudocatenulatum* and *Roseburia intestinalis* (*P*_interaction_ = 0.09).

**Conclusions:**

Our study provides the first large-scale evidence supporting taxonomic and functional shifts of the gut microbiome in individuals with asymptomatic diverticulosis. The suggestive interaction between gut microbiota and BMI on prevalent diverticulosis deserves future investigations.

**Supplementary Information:**

The online version contains supplementary material available at 10.1186/s13073-024-01374-9.

## Background

Colonic diverticulosis, the presence of outpouchings from the colonic lumen, is the most commonly reported lesion found on routine colonoscopy, affecting more than 50% of individuals aged 60 years and above [1]. Although most cases of diverticulosis are asymptomatic, about 25% will become symptomatic [2] and up to 4% develop complications, including diverticulitis and diverticular bleeding [3]. Diverticular complications account for nearly 2 million ambulatory visits, 208,015 hospital admissions, and 4602 deaths every year in the United States, imposing a significant burden on patients and the US health care system [4].

The pathogenesis of diverticulosis is largely unknown with a growing body of evidence challenging several previously proposed mechanisms including the prevailing hypotheses of fiber deficiency [5–10] and chronic inflammation [11, 12]. Recent genome-wide association studies have shed some insight into critical biological pathways including those involved in neuromuscular homeostasis, connective tissue, and intestinal motility [13, 14]. There is also emerging evidence suggesting that the gut microbiota may play an important role in the pathophysiology of diverticular diseases (reviewed in [2]). This relationship is largely thought to be related to the possible role of the gut microbiome in regulating integrity of the intestinal epithelial barrier function [12] and gut motility [14, 15]. Prior studies suggest that individuals with symptomatic uncomplicated diverticular disease (SUDD) are more likely to have reduced relative abundance of taxa with a possible anti-inflammatory effect, such as *Clostridium cluster IV* [12], and overgrowth of *Akkermansia* [16, 17]. However, the role of gut microbiota in early stages of the disease, specifically in asymptomatic diverticulosis, is unclear. We therefore analyzed the fecal microbiota of individuals with and without asymptomatic diverticulosis from a colonoscopy-based cohort, the **G**astro **I**ntestinal **D**isease and **E**ndoscopy **R**egistry (GIDER) using shotgun metagenomic sequencing to characterize microbial taxonomic composition and metabolic functions.

## Methods

### Study population

Our study population included participants in GIDER with available stool sample collected prior to the screening colonoscopy from 2018 to 2021. The detailed protocol for GIDER has been described previously [18]. Briefly, GIDER is a colonoscopy-based longitudinal cohort of individuals older than 18 years at Massachusetts General Hospital (MGH) that was established to identify biomarkers that are associated with gastrointestinal conditions. Prior to scheduled screening or surveillance colonoscopy, study participants were invited to complete a baseline questionnaire to assess diet, lifestyle factors, medications, and medical history. Stool, saliva, and urine samples were collected prior to colonoscopy. Participants are followed through annual questionnaires where information on diet, lifestyle, medical diagnoses, and medications are collected and updated. For this study, we excluded individuals with a history of gastrointestinal cancer, hereditary non-polyposis colorectal cancer, familial adenomatous polyposis, inflammatory bowel disease, colorectal surgery and bleeding disorders, and those who had used antibiotics in the 2 months before the scheduled colonoscopy. Additionally, we excluded participants with history of possible complications from diverticulosis including diverticulitis and lower gastrointestinal bleed, and those with chronic abdominal pain that may represent symptomatic diverticulosis. After exclusion, 752 people with available stool samples prior to colonoscopy were eligible for our study. All study participants provided informed consent. Our study was approved by Partners Human Research Committee, the Institutional Review Board of Mass General Brigham. All participants provided written informed consent before enrollment.

### Outcome ascertainment

Diverticulosis was confirmed during colonoscopy. All colonoscopies were performed by board-certified gastroenterologists. A study coordinator obtained information on the presence and location of diverticula from the endoscopist at the end of each procedure. In a validation study, we compared the correlation between colonoscopy and computerized tomography (CT) scan for diagnosing diverticulosis. We identified 173 GIDER study participants who underwent an abdominal CT scan within 6 months of their colonoscopy. A board-certified radiologist who was blinded to colonoscopy-based diagnoses of diverticulosis reviewed the CT reports and images to make a diagnosis. The demographic characteristics of these individuals according to diverticulosis diagnosis based on CT were included in Additional file 2, Table S1. The sensitivity and specificity of colonoscopy-based diagnosis against abdominal CT were 67% and 98%, respectively, and the kappa coefficient was 0.63. Participants who did not have diverticulosis on colonoscopy were used as controls.

### Stool sample collection and processing

Participants self-collected stool samples using a collection kit containing ethanol. Stool samples were handled at ambient temperature and stored in − 80 °C freezers upon arrival at MGH. Genomic DNA was extracted from the stool samples using QIAGEN AllPrep DNA/RNA Mini Kit (Valencia, CA, USA). Stool self-collection and DNA extraction from stool aliquots were performed according to the Human Microbiome Project (HMP) protocols [19].

### Metagenomic sequencing and profiling

Metagenomic sequencing libraries were prepared using the Nextera XT DNA Library Preparation Kit (Illumina) according to the manufacturer’s recommended protocol. Metagenomic sequencing was performed at the Broad Institute (Cambridge, MA) using the Illumina HiSeq 2500 platform. Raw sequencing reads were processed using the KneadData pipeline version v0.8.0 (http://huttenhower.sph.harvard.edu/kneaddata) as part of the bioBakery 3, a suite with integrated methods for metagenomic data processing [20]. Default parameters were used to trim short reads (< 50% of total input read length) and remove sequences of human origin. Metagenomic sequencing and processing of all samples were performed in three different batches. Taxonomic and functional profiling were conducted using the bioBakery 3 meta’omics workflow according to the Human Microbiome Project protocol [20]. Specifically, we used the Metagenomic Phylogenetic Analysis tool (MetaPhlAn version 3.0.6) to classify metagenomic sequencing reads to taxonomies and generate the relative abundances in each sample [21]. Metagenomics functional profiling was performed using HMP Unified Metabolic Analysis Network (HUMAnN version 3.0.0) tool [22]. Briefly, based on identified microbial species from taxonomic profiling for each sample, reads are recruited to construct a sample-specific database of pangenomes of all species identified [23]. Unmapped reads were aligned against a comprehensive protein database UniRef90 [24] using translated search [25]. These alignments were processed in a species-specific manner and were weighted by quality and sequence length to estimate gene family abundance. Finally, gene families annotated to metabolic reactions were further analyzed to reconstruct and quantify metabolic pathways in each sample based on MetaCyc [26].

### Assessment of covariates

We collected the demographic and lifestyle factors through a baseline questionnaire. Dietary intake was assessed using a brief dietary questionnaire. Specifically, prior to colonoscopy, participants were asked to report the frequency of food consumption on an eight-category scale (never, once/week, 2–4 times/week, 5–6 times/week, once/day, 2–3 times/day, 4–5 times/day, > 6 times/day). Our analysis focused on the consumption of fruit, vegetables, red meat, and processed meat. We categorized fruit and vegetable intake into less than once/day, 1–2 times/day, > 2 times/day, and further grouped red and processed meat intake into ≤ 1/week, 2–4 times/week, and > 4 times/week. Dietary pattern was grouped according to self-reported dietary preferences with respect to meat and included “standard diet,” “standard diet with limited red meat (< 3 times/week),” “standard diet with poultry/fish (no red meat),” and “vegetarian/vegan.” This brief dietary questionnaire was validated against the Semiquantitative Food Frequency Questionnaire (SFFQ) [27] in our previous study [18]. In addition to dietary factors, we also calculated body mass index (BMI) based on participants’ self-reported height and weight at baseline. Smoking was categorized into never, former, and current smokers. Physical activity was assessed by asking individual’s average time spent per week on various recreational activities using a previously validated questionnaire [28]. We assigned a metabolic equivalent task (MET) to each activity based on previously established guidelines [29] and determined the total amount of MET hours on average per week across all activities. Regular use of medication was defined as use greater than twice per week. Use of probiotics in the past 2 months and use of antibiotics in the last year were also ascertained.

### Statistical analyses

To estimate the proportion of variation in the disease status of asymptomatic diverticulosis explained by individual factors, we first used Poisson regression to model the association between each individual factor and prevalence of diverticulosis. For gut microbiome, we first performed principal component (PC) analysis to reduce the dimension of the microbial taxonomic features and used the first 111 PCs that explained 90% variance of the microbiome composition in the Poisson regression model. We then calculated the adjusted *R*-squared measures to account for over- or under-dispersion [30, 31]. To evaluate broad differences in the microbiome composition, we calculated alpha diversity using the Chao1 index based on taxonomic profiling results of the microbial species for each sample and compared the average alpha diversity between cases and controls using non-parametric Wilcoxon rank sum tests. In order to determine variability in the taxonomic composition at the species level, we calculated Bray-Curtis dissimilarity. We performed a permutational multivariate analysis of variance (PERMANOVA) of Bray-Curtis dissimilarities (999 permutations) to quantify the proportion of variation in the microbial taxonomy explained by demographic factors (age, sex, race, and ethnicity), disease status (cases vs controls), lifestyle factors (dietary factors, smoking, BMI, and physical activity), and medication use (non-steroidal anti-inflammation drugs [NSAIDs], antibiotics, and probiotics).

For per-feature analyses, we evaluated the differences in the relative abundance of taxonomic species and metabolic pathways between asymptomatic diverticulosis cases and controls using the multivariable linear mixed model (MaAsLin2 version 1.4.0 http://huttenhower.sph.harvard.edu/maaslin2) [32]. All models were adjusted for age at colonoscopy, sex, BMI, and dietary patterns (standard diet, limited red meat, no read meat, or vegetarian/vegan), selected based on their known association with diverticulosis and gut microbiome composition and function, as fixed effects and sequencing batch as random effects:

$$\mathrm{microbiome}\;\mathrm{features}\hspace{0.17em}\sim\hspace{0.17em}\mathrm{diverticulosis}\;(\mathrm{yes}/\mathrm{no})\hspace{0.17em}+\hspace{0.17em}\mathrm{age}\hspace{0.17em}+\hspace{0.17em}\mathrm{sex}\hspace{0.17em}+\hspace{0.17em}\mathrm{BMI}\hspace{0.17em}+\hspace{0.17em}\mathrm{dietary}\;\mathrm{pattern}\hspace{0.17em}+\hspace{0.17em}(1\;\vert\;\mathrm{batch})$$

We used the Benjamini-Hochberg false discovery rate (FDR) to correct for multiple comparisons. An FDR-corrected *p* value (*q* value) < 0.25 was considered statistically significant, in line with prior discovery-based microbiome studies [33, 34]. In sensitivity analysis, we additionally adjusted for the use of probiotics in the past 2 months and the use of antibiotics in the last year as confounders in the multivariable linear mixed model.

To examine whether the associations between asymptomatic diverticulosis and previously identified risk factors (i.e., age at colonoscopy, sex, BMI, smoking status, physical activity, and dietary factors) were modified by gut microbial compositions, we built Poisson regression models that simultaneously include the main effects of the risk factor and the principal component loading score 1 (PCo1) or the relative abundance of a microbial species, as well as the product term of the two main effects in addition to other confounders. We used two-sided likelihood ratio test by comparing models with and without the interaction term to calculate *P*_interaction_. We also conducted secondary analysis comparing the differences in taxonomic structure and metagenomic functions between cases and controls, stratified by the anatomic site (right vs left colon) of the diverticula.

## Results

**Fig. 1:**
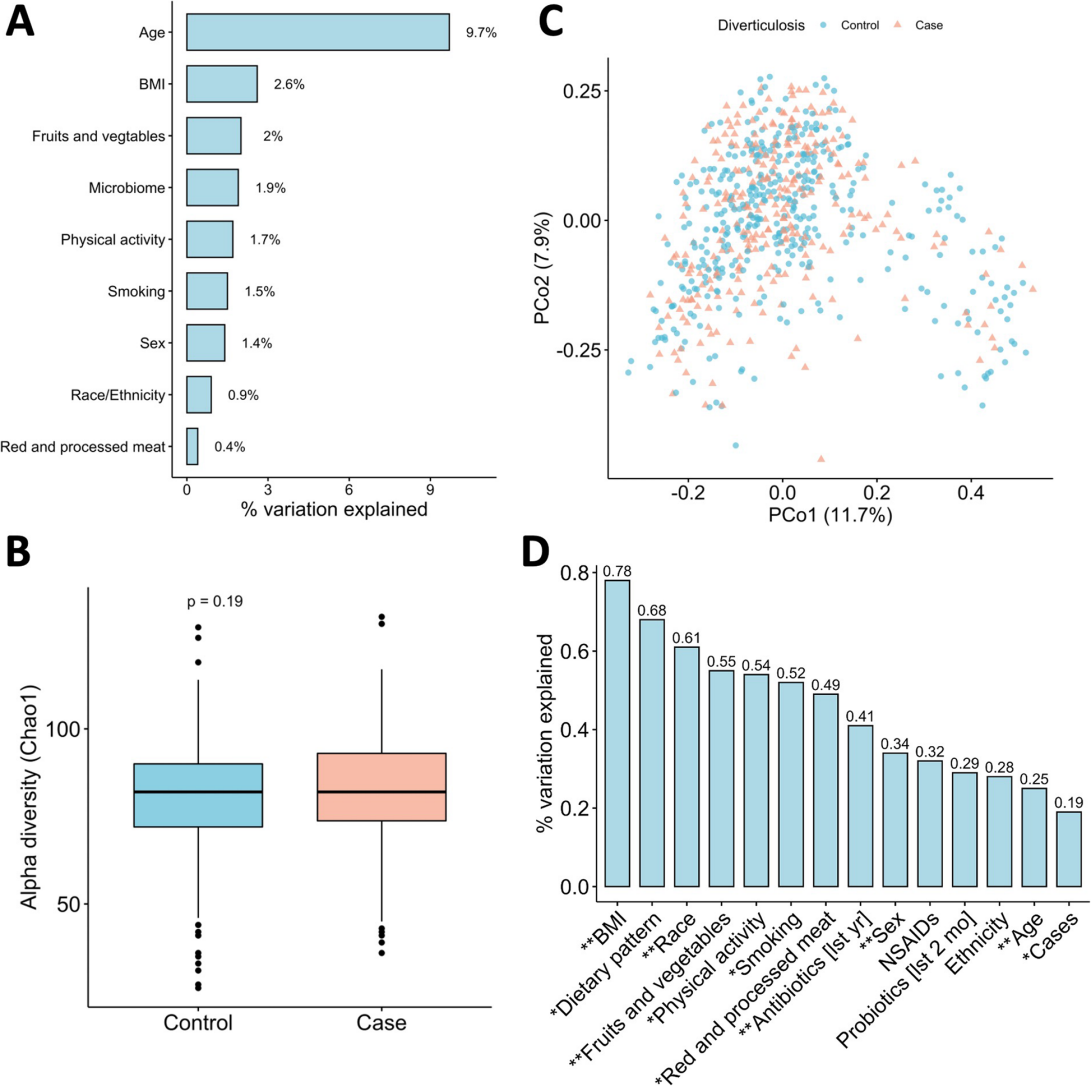
**A** Variation in the disease status of asymptomatic diverticulosis explained by individual factors; **B** alpha diversity (Chao1 index) between asymptomatic diverticulosis and controls; *p* value was calculated using non-parametric Wilcoxon rank sum test; **C** principal coordinates analysis (PCoA) of study participants based on Bray-Curtis distances between gut metagenomic species profiles; **D** the proportion of variation in the microbial taxonomy explained by individual factor based on permutational multivariate analysis of variance (PERMANOVA) of the Bray-Curtis dissimilarity matrix. Exact *R*^2^ and *p* values are included in Additional file 2, Table S2. BMI, body mass index; NSAIDs, non-steroidal anti-inflammatory drugs

We included a total of 684 participants from 752 GIDER participants who had metagenomic data in our study. Among these participants, 284 (42%) were found to have diverticulosis during colonoscopy. We observed that on average those with diverticulosis were older (mean age at colonoscopy: 65 vs 58 years), more likely to be male, obese, and former smokers and less likely to be physically active (> 21 MET hours/week) or regularly consume fruit and vegetables (> 2 times/day, Table 1). We observed that age explained more than 9% variation in the disease status of asymptomatic diverticulosis whereas gut microbiome composition explained 1.9% variation (Fig. 1A).

**Table 1:** Characteristics of participants in GIDER according to presence of diverticulosis

**Characteristics**	**Total**	**Case**	**Control**
	**(*N* = 684)**	**(*N* = 284)**	**(*N* = 400)**
**Age at colonoscopy (years), mean (SD)**	61 (10)	65 (8.8)	58 (9.8)
< 50	57 (8)	8 (3)	49 (12)
50–59	248 (36)	71 (25)	177 (44)
60 +	379 (55)	205 (72)	174 (44)
**Female, *n* (%)**	364 (53)	129 (45)	235 (59)
**Race, *n* (%)**			
Asian	20 (3)	4 (1)	16 (4)
Black	25 (4)	7 (2)	18 (4)
White	630 (92)	270 (95)	360 (90)
With 1 + race unknown	9 (1)	3 (1)	6 (2)
**Hispanic, *n* (%)**			
Yes	21 (3)	8 (3)	13 (3)
No	660 (96)	275 (97)	385 (96)
Missing	3 (0.4)	1 (0.4)	2 (0.5)
**BMI (kg/m**^**2**^**), mean (SD)**	27 (5.5)	28 (5.7)	26 (5.2)
Underweight	14 (2)	3 (1)	11 (3)
Normal	236 (35)	74 (26)	162 (40)
Overweight	239 (35)	109 (38)	130 (32)
Obese	154 (23)	79 (28)	75 (19)
Missing	41 (6)	19 (7)	22 (6)
**Smoking, *n* (%)**			
Never	269 (64)	80 (52)	189 (70)
Former	114 (27)	55 (36)	59 (22)
Current	10 (2)	5 (3)	5 (2)
Missing	30 (7)	13 (8)	17 (6)
**Physical activity (MET hours/week), mean (SD)**	38 (39)	35 (45)	40 (34)
< 7.5	106 (15)	60 (21)	46 (12)
7.5–20.9	162 (24)	69 (24)	93 (23)
21 or more	379 (55)	136 (48)	243 (61)
Missing	37 (5)	19 (7)	18 (5)
**Antibiotics use in the last year, *n* (%)**			
No	489 (71)	190 (67)	299 (75)
Yes	138 (20)	65 (23)	73 (18)
Missing	57 (8)	29 (10)	28 (7)
**Probiotics use in the last 2 months, *n* (%)**			
No	545 (80)	228 (80)	317 (79)
Yes	91 (13)	30 (11)	61 (15)
Missing	48 (7)	26 (9)	22 (6)
**NSAIDs use, *n* (%)**			
No	459 (67)	177 (62)	282 (70)
Yes	172 (25)	80 (28)	92 (23)
Missing	53 (8)	27 (10)	26 (7)
**Dietary pattern, *n* (%)**			
Standard diet	361 (53)	149 (52)	212 (53)
Standard diet with limited red meat (< 3 times/week)	209 (31)	84 (30)	125 (31)
Standard diet with poultry/fish (no red meat)	51 (7)	22 (8)	29 (7)
Vegetarian/vegan	23 (3)	9 (3)	14 (4)
Missing	40 (6)	20 (7)	20 (5)
**Fruit and vegetable consumption**			
**Mean (SD), servings per week, *n* (%)**	**21 (17)**	**22 (17)**	**22 (17)**
< 1 times/day	123 (18)	62 (22)	61 (15)
1–2 times/day	172 (25)	84 (30)	88 (22)
> 2 times/day	279 (41)	95 (33)	184 (46)
Missing	110 (20)	43 (20)	67 (20)
**Red and processed meat consumption**			
**Mean (SD), servings per week, *n* (%)**	**3.7 (3.4)**	**4.0 (3.9)**	**3.5 (2.9)**
≤ 1 time/week	147 (21)	62 (22)	85 (21)
2–4 times/week	243 (36)	96 (34)	147 (37)
> 4 times/week	141 (21)	67 (24)	74 (18)
Missing	153 (20)	59 (20)	94 (20)

*Abbreviations*: *BMI* body mass index, *GIDER* GastroIntestinal Disease and Endoscopy Registry, *NSAID* non-steroidal anti-inflammatory drug, *MET* metabolic equivalent, *SD* standard deviation

For the overall composition of the gut microbiome, we observed no significant differences in the alpha diversity (Chao1 index) between diverticulosis and controls (Wilcoxon rank sum *p* value = 0.19, Fig. 1B). The principal coordinate analysis of the species-level Bray-Curtis dissimilarity also demonstrated that disease status was not a major driver of the overall structural variation of the gut microbiome (Fig. 1C). This was further supported by the permutational multivariate analysis of variance (PERMANOVA), where we observed that disease status explained only 0.2% variance of the gut microbial community (*p* value = 0.01, Fig. 1D, Additional file 2, Table S2). BMI, dietary pattern, race, and fruit/vegetable consumption were the top factors that explained most of the variation of gut microbiome composition, ranging from 0.5 to 0.8%.

**Fig. 2:**
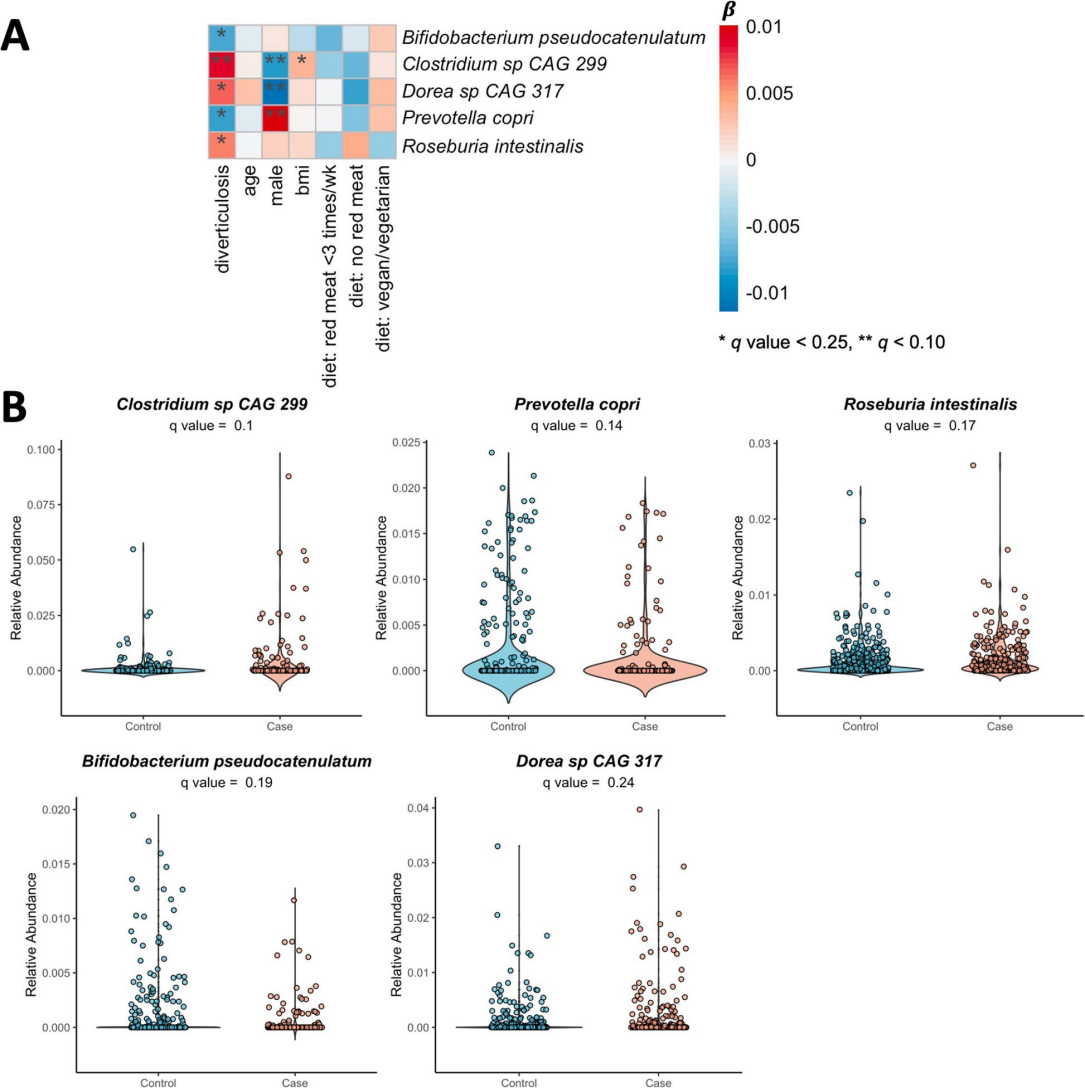
Microbial species and asymptomatic diverticulosis. **A** Association between the relative abundance of fecal microbial species and asymptomatic diverticular diseases adjusted for age at colonoscopy, sex, body mass index (BMI), and dietary patterns (limited red meat, no read meat, or vegetarian/vegan vs standard diet) using the multivariable linear mixed model. **B** Relative abundances of microbial species significantly associated with asymptomatic diverticulosis (*q* value < 0.25)

For per-feature analyses of the relative abundance of taxonomic species, we included a total of 169 gut microbial species after filtering (minimum prevalence [> 10%] and relative abundance [> 0.01%]). Five species were significantly different in abundance between diverticulosis and controls (*q* values < 0.25; Fig. 2 and Additional file 2, Table S3). *Roseburia intestinalis*, *Dorea *sp. CAG:317, and *Clostridium *sp. CAG:299 were significantly enriched in asymptomatic diverticulosis (*q* values = 0.17, 0.24, and 0.10, respectively). Previous reports have shown that relative abundance of *R. intestinalis* is associated with higher consumption of meat [35], while it is inversely associated with dietary fiber and Healthy Eating Index (HEI) [35, 36]. Although *Clostridium *sp. CAG: 299 is uncharacterized, several *Clostridium* species were previously linked with western-style diets and red meat intake [37]. In contrast, controls had higher abundance of *Bifidobacterium pseudocatenulatum* (*q* value = 0.19), an anaerobic bacterium positively associated with healthy plant diet index and higher fiber intake [35, 36], and *Prevotella copri* (*q* value = 0.14), which was shown to be associated with improved glucose metabolism [38]. *P. copri-*produced succinate was demonstrated to be actively involved in intestinal gluconeogenesis in animal study and was associated with improved glycemic control [39]. Additional adjustment for the use of antibiotics and probiotics in the multivariable mixed linear models did not  significantly change the magnitude of associations for *R. intestinalis*, *Clostridium *sp. CAG:299, *P. copri*, *Dorea *sp. CAG:317, and *B. pseudocatenulatum *(Additional file 2, Table S4); however, the latter two were not statistically significant (*q* values = 0.26 and 0.31, respectively). This analysis also identified higher abundance of *Gordonibacter pamelaeae* and *Parabacteroides johnsonii* (*q* values = 0.24 for both, Additional file 1, Fig. S1; Additional file 2, Table S4) in diverticulosis as compared to controls. *Parabacteroides johnsonii* has been associated with higher levels of circulating C-reactive protein in a previous study [36].

**Fig. 3:**
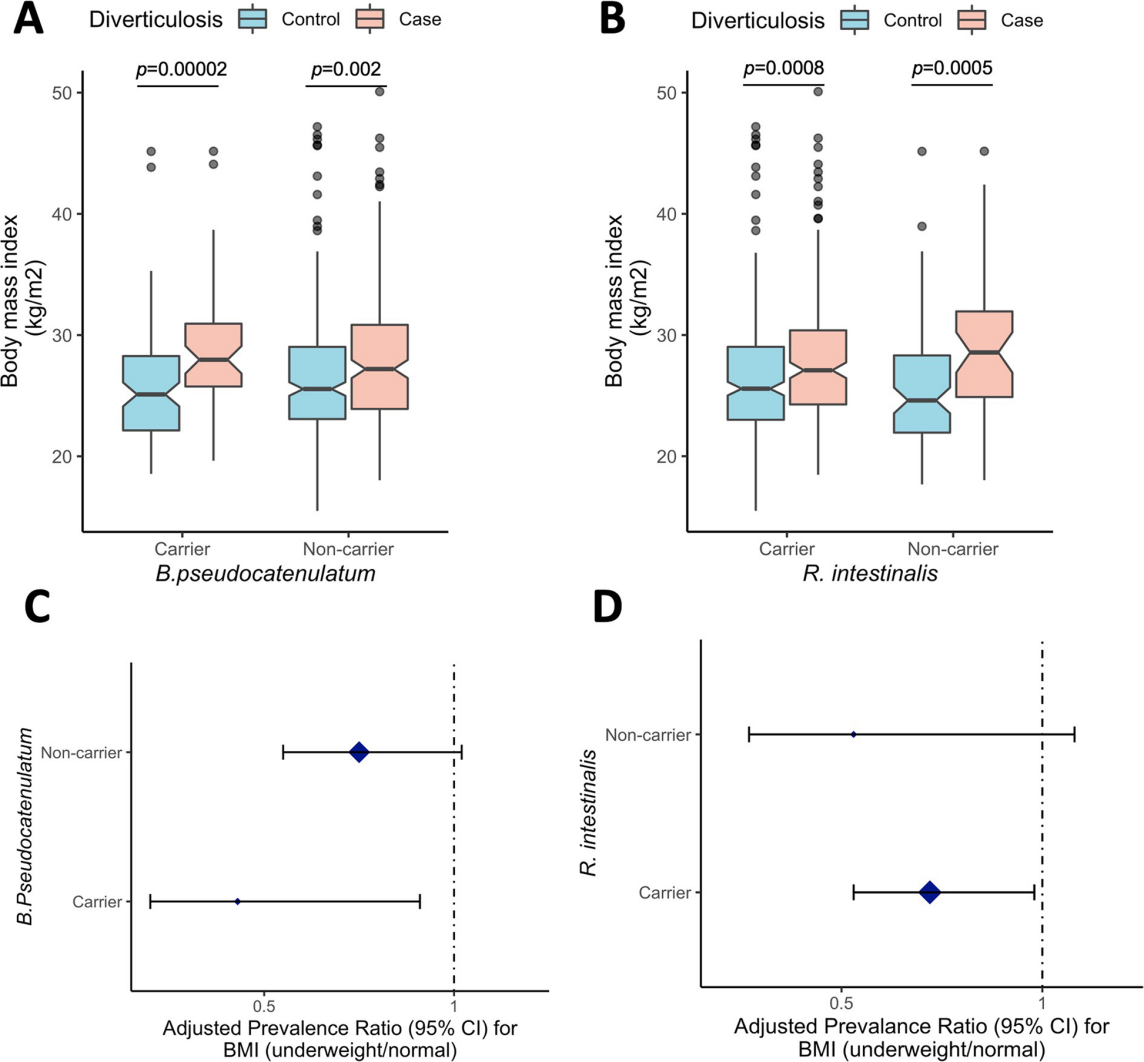
Distribution of BMI between asymptomatic diverticulosis and controls according to the status of **A** *Bifidobacterium pseudocatenulatum* (carriers *n* = 159, non-carriers *n* = 525) and** C** *Roseburia intestinalis* (carriers *n* = 544, non-carriers *n* = 140). *p* values were calculated using non-parametric Wilcoxon rank sum test. Adjusted prevalence ratio and 95% CI between BMI (underweight/normal weight) with the prevalence of asymptomatic diverticulosis according to the presence and absence of **B** *Bifidobacterium pseudocatenulatum* and **D** *Roseburia intestinalis* were calculated using Poisson regression model adjusted for age, sex, and dietary pattern (standard diet, limited red meat, no red meat, or vegetarian/vegan)

In our exploratory analyses, we evaluated whether known associations between lifestyle risk factors (i.e., BMI, dietary fiber intake, physical activities) and asymptomatic diverticulosis differ according to gut microbial profiles. We observed that underweight/normal BMI was associated with lower prevalence of asymptomatic diverticulosis, which was only evident among *B. pseudocatenulatum* carriers (prevalence ratio (PR) = 0.43, 95% CI = 0.20–0.91, *p* value = 0.03) and *R. Intestinalis* carriers (PR = 0.72, 95% CI = 0.53–0.98, *p* value = 0.04), but not among their non-carrier counterparts (*B. pseudocatenulatum* non-carriers: PR = 0.75, 95% CI = 0.55–1.02, *p* value = 0.07; *R. intestinalis* non-carriers: PR = 0.53, 95% CI = 0.28–1.08, *p* value = 0.08, Fig. 3), although these interactions did not reach statistical significance (both *P*_interaction_ = 0.09). We did not observe any evidence of effect modifications between other demographic and lifestyle risk factors (age, sex, smoking, dietary pattern, and physical activity) and the five significant microbial species on prevalent diverticulosis.

**Fig. 4:**
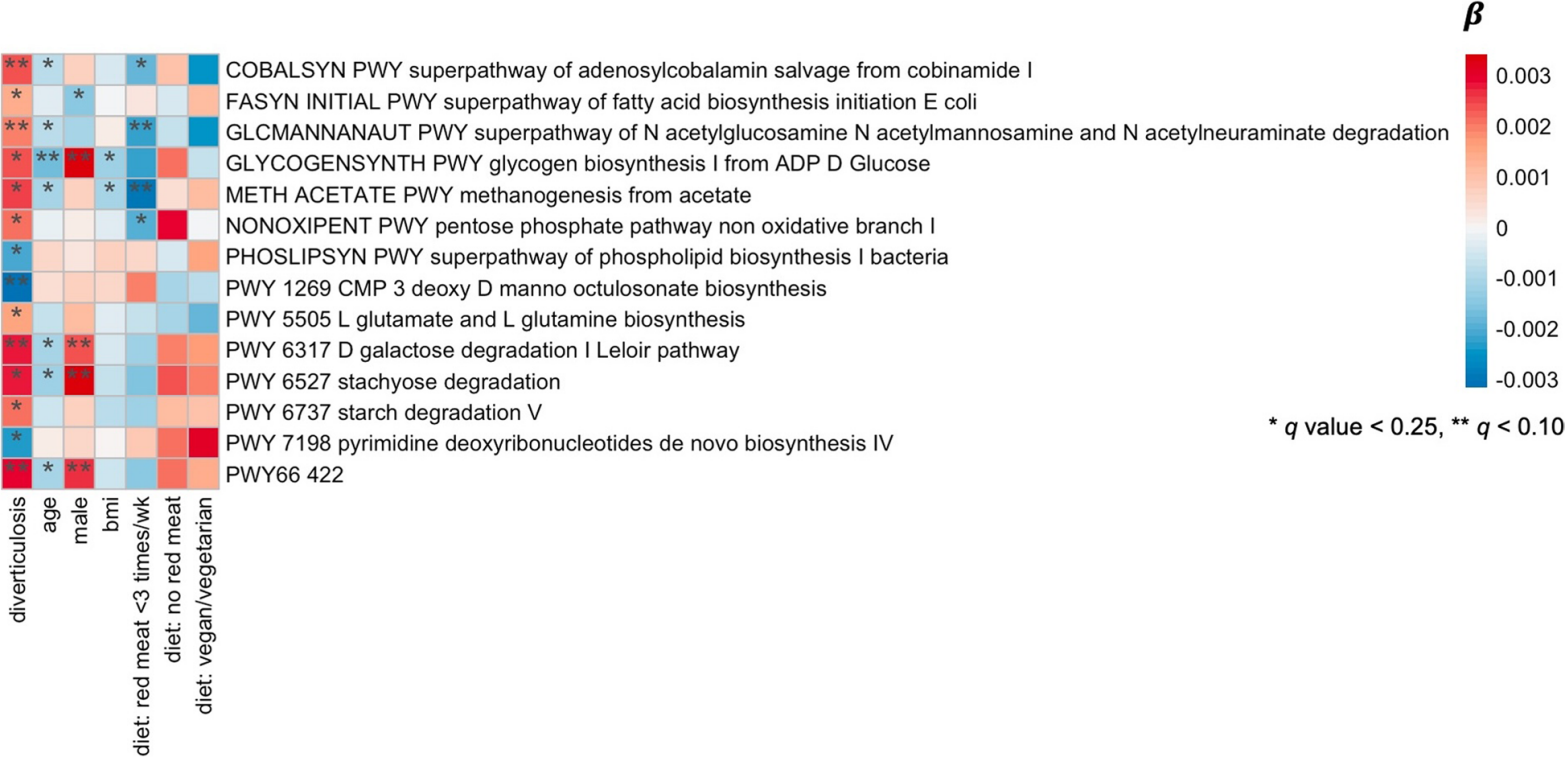
Metabolic pathways significantly associated with asymptomatic diverticulosis adjusted for age at colonoscopy, sex, body mass index (BMI), and dietary patterns (standard diet, limited red meat, no read meat, or vegetarian/vegan) using the multivariable linear mixed model. All *q* values were derived from the multivariable-adjusted models and corrected for multiple comparison using the Benjamini-Hochberg false discovery rate (FDR) method

For functional potential of the gut microbial communities, estimated as the relative abundance of metabolic pathways, a total of 228 metabolic pathways were included after filtering (minimum prevalence [> 10%] and relative abundance [> 0.1%]). We observed that controls had significant enrichment of microbial function for common housekeeping processes, such as the biosynthesis of acid sugar 3-deoxy-α-D-manno-2-octulosonate, which is a component of bacterial lipopolysaccharides (PWY 1269, *q* value = 0.07, Fig. 4 and Additional file 2, Table S5). In contrast, several microbial functions were enriched in individuals with asymptomatic diverticulosis, including metabolic pathways involved in the biosynthesis of vitamin B12 (COBALSYN PWY, *q* value = 0.03), glycolysis-related pathways (PWY 66–422, *q* value = 0.05; PWY 6317, *q* value = 0.04), and a pathway involved in the degradation of amino sugars (GLCMANNANAUT PWY, *q* value = 0.08, Fig. 4 and Additional file 2, Table S5). In sensitivity analyses, we additionally adjusted for the use of antibiotics and probiotics. We found that the magnitude of association between metabolic pathways and asymptomatic diverticulosis remained similar, except for three metabolic pathways which were no longer statistically significant (PWY 7198, *q* value = 0.26; PWY 5505, *q* value = 0.34; PHOSLIPSYN PWY, *q* value = 0.34, Additional file 2, Table S6).

Due to the significant variation in the location of diverticula within the colon across different regions of the world [40], we further examined the distribution of right- vs left-diverticulosis and explored compositional and functional differences in the gut microbiome between right- and left-diverticulosis and controls. In our study population, 201 out of 284 (71%) diverticulosis cases were left-sided, consistent with reports from the western countries [41, 42]. Comparing controls with right- and left-sided diverticulosis, we observed that individuals with right-sided diverticulosis had the highest alpha diversity, although this comparison did not reach statistical significance (Additional file 1, Fig. S2A). Principal coordinate analysis based on species-level Bray–Curtis dissimilarity suggested that disease location was not the main driver of the variation of the gut microbial communities (Additional file 1, Fig. S2B). In per-feature analysis, although there was no significant difference of the relative abundances of gut microbial species and their metabolic functions between left- vs right-sided diverticulosis, five microbial species, including *P. copri*, were significantly different in relative abundance between left-sided diverticulosis and controls (*q* value < 0.25, Additional file 1, Fig. S3A; Additional file 2, Table S6). Several metabolic pathways involved in glycolysis and carbohydrate metabolism remained enriched in individuals with left-sided diverticulosis, whereas microbial functions involved in five metabolic pathways, such as amino acid and carbohydrate metabolism, were enriched in right-sided diverticulosis compared to controls (see Additional file 1, Fig. S4; Additional file 2, Table S7).

## Discussion

In a colonoscopy-based cohort, we showed unique differences between the fecal microbiota of individuals with diverticulosis as compared to those without even after adjustment for important lifestyle risk factors. These differences included higher relative abundance of *Roseburia intestinalis* and lower relative abundance of *Bifidobacterium pseudocatenulatum* and *P. copri* as compared to controls.

Although there is emerging evidence on the relationship between the fecal microbiome and diverticular disease, most prior studies have focused on symptomatic disease or diverticulitis [17, 43, 44]. Studies evaluating specific microbial species in association with asymptomatic diverticulosis are limited. A case-control study of middle-aged female participants reported no differences of the total number of bacteria in the fecal samples between 13 individuals with asymptomatic diverticulosis and 16 controls based on RT-PCR profiling of targeted microorganisms [16]. It also observed significantly higher abundance of *Akkermansia muciniphila* in individuals with asymptomatic diverticulosis compared to controls. In addition, Barbara et al. used a 16S rRNA profiling approach and found that the overall fecal microbiota distribution was similar between 16 individuals with asymptomatic diverticulosis and 14 controls [12]. However, they did not observe any significant difference in the relative abundance of bacteria between the two groups. Both studies supported our observation that the overall fecal microbial structure is not significantly different between diverticulosis and controls. However, these prior studies were limited by small sample size or lower resolution of microbiome profiling (i.e., PCR or 16S rRNA). More recently, a study of 19 individuals with asymptomatic diverticulosis and 24 controls found no differences in microbiota richness or diversity based on mucosal biopsies obtained from both sigmoid and transverse colon [45]. This was further supported by a population-based study by Alexandersson et al. which observed no differences in richness, diversity, or taxonomy composition of both mucosa-associated and fecal microbiota between individuals with and without diverticulosis [46]. Lastly, a large colonoscopy-based study led by Jones and colleagues included 226 individuals with asymptomatic diverticulosis and 309 diverticula-free controls [47]. Using 16S rRNA profiling to characterize mucosa-associated microbiota, the authors found minimal differences in the alpha diversity and richness between individuals with and without diverticula, with no significant differences in Bray-Curtis dissimilarity or microbial composition. Therefore, our study that leverage metagenome sequencing to generate detailed information on composition and function of the fecal microbiome while also collected detailed information on lifestyle factors in a large colonoscopy-based cohort significantly expand on these prior work.

Several gut microbial species that were associated with asymptomatic diverticulosis have been linked to dietary factors in previous reports. Specifically, *Bifidobacterium pseudocatenulatum*, the microbial species significantly enriched in controls in our study, is an anaerobic bacterium positively associated with healthy plant diet index and higher fiber intake in recent large-scale population-based studies [35, 36]. Similarly, *Roseburia intestinalis*, an anaerobe that we found to be significantly more abundant in participants with asymptomatic diverticulosis, was previously linked with higher consumption of meat [35] and inversely associated with dietary fiber and Healthy Eating Index (HEI) [35, 36]. These results are in line with previous epidemiologic studies that have shown consumption of fruit and vegetables and higher fiber intake are associated with lower prevalence of diverticulosis [6–8, 18], while increased consumption of red meat is associated with a higher prevalence [7, 48].

Previous studies found that obesity was associated with a higher prevalence of asymptomatic diverticulosis after adjustment for diet [49–52]. Here we show a suggestive interaction between obesity and microbial species in association with prevalent diverticulosis. Specifically, the association between obesity and diverticulosis was only evident among *B. pseudocatenulatum* and *R. intestinalis* carriers. Interestingly, previous work has demonstrated that ingestion of the *B. pseudocatenulatum* strain CECT 7765 in high-fat diet-fed obese mice led to lower levels of serum cholesterol and triglycerides, reduced obesity-associated systemic inflammation, and improved insulin resistance and glucose tolerance [53]. Taken together, our results suggest that the association between obesity and diverticulosis may be modified by unique features in the gut microbiota.

In our study population, *P. copri* was present in 19% of participants and was shown to be inversely associated with asymptomatic diverticulosis, particularly left-sided diverticulosis. The direct effect of *P. copri* on human health is still largely unknown. Some studies reported its positive association with inflammatory diseases such as rheumatoid arthritis [54] and ankylosing spondylitis [55], while others demonstrated that *P. copri* was associated with improved glucose metabolism [38] and insulin tolerance [39] in response to fiber-rich diet. This discrepancy is likely explained, at least in part, by the heterogeneity in different strains of *P. copri* as well as their varying prevalence between western and non-westernization populations [56]. Our observation that *P. copri* was associated with a lower prevalence of diverticulosis may be explained partially by its role as a dietary fiber-degrader and associated effect on improved glucose metabolism. Given the observational nature of our study, additional investigation is needed to further identify the specific subspecies of *P. copri* and corresponding effect on modulating dietary factors in the pathogenesis of diverticulosis.

We also found that higher abundance of *Clostridium *sp. CAG: 299, an uncharacterized species, is positively associated with prevalent diverticulosis. Recent large-scale population-based studies reported several *Clostridium* species to be associated with unhealthy dietary patterns [35, 57]. In addition, these species were inversely associated both recent and long-term dietary fiber intake [36]. The metabolic pathway involved in the degradation of amino sugars (GLCMANNANAUT PWY), known to be possessed by several *Clostridium* species, was also enriched in asymptomatic diverticulosis. Together, our findings support previous observations of an inverse association between healthy diet, particularly dietary fiber, and asymptomatic diverticulosis. Our data also suggests several important gut microbial species, including *B. pseudocatenulatum* and *R. intestinalis* that may interact with lifestyle factors in the pathogenesis of diverticulosis.

Our additional functional analyses based on microbial-specific genes revealed that several metabolic pathways were significantly different between individuals with diverticulosis and controls. Specifically, microbial involvement in the degradation pathway of N-acetyl-glucosamine (GLCMANNANAUT PWY) was significantly enriched in asymptomatic diverticulosis. This finding aligns with the study conducted by Tursi et al., where a fecal metabolome assessment indicated that individuals with asymptomatic diverticulosis had significantly lower levels of N-acetyl-glucosamine compared to health controls [16]. Taken together, these results suggest that the enrichment of the microbial pathway involved in the degradation of N-acetyl-glucosamine likely contributes to lower levels of this metabolite in individuals with diverticulosis. Further research is needed to elucidate the association between the decrease in N-acetyl-carbohydrates and specific microbial species, and to understand the mechanisms underlying this association with the presence of diverticula.

There are some limitations to be considered when interpreting our study findings. First, our study is cross-sectional by design. The gut microbiome was measured at the time of colonoscopy/diagnosis. Therefore, we cannot establish temporality of the associations based on our study design. Second, due to the observational nature of our study, we cannot exclude residual confounding. Nevertheless, we adjusted for known and putative risk factors for diverticulosis in our analyses. Third, we note that colonoscopy is not the gold standard for diagnosing diverticulosis and therefore there may have been misclassification of outcome in our study. However, in our validation study, the sensitivity and specificity of colonoscopy as compared to CT scan were nearly 70% and 100%, respectively. Also future studies may consider using Diverticular Inflammation and Complication Assessment (DICA) endoscopic classification, which has previously been shown to accurately predict outcome of diverticular disease [58]. Fourth, we had limited dietary data and therefore could not estimate specific nutrient intake such as fiber or fat consumption. Lastly, despite our study representing the largest study to date, our sample size may have not be large enough for our secondary analysis by anatomic location or to identify more modest associations and explore for potential effect modification and mediation by diet and BMI.

Our study also has several strengths. We leveraged shotgun metagenomic sequencing which significantly expands on prior studies and provides more in-depth and generalizable characterization of the gut microbiome. In addition, we used a well-characterized cohort with detailed demographic and lifestyle factors and therefore were able to account for potential confounders of the association between fecal microbiome and diverticulosis.

## Conclusions

In a large colonoscopy-based cohort, we showed that relative abundance of several microbial species that have previously been linked to diet were significantly different in individuals with asymptomatic diverticulosis as compared to controls. Our data also point to a possible relationship between BMI, the gut microbiota, and prevalent diverticulosis. Future studies should explore the intriguing interaction between gut microbiota, obesity, and diet in the pathogenesis of diverticulosis.

### Supplementary Information

Additional file 1: Supplementary results and Figs. S1–S4. Fig. S1 Microbial species and asymptomatic diverticulosis—sensitivity analysis. Fig. S2 A) Alpha diversitybetween asymptomatic diverticulosis at right colon, left colon, versus healthy controls; B) Principal coordinates analysisof study participants based on Bray-Curtis distances between gut metagenomic species profiles. Fig. S3 Taxonomic profiles of the gut microbiome significantly associated with asymptomatic diverticulosis according to anatomic sites. Fig. S4 Metabolic pathways and diverticulosis according to anatomic sites

Additional file 2: Tables S1–S8. Table S1 Demographic characteristics of individuals in the validation study comparing the diagnosis of diverticulosis based on colonoscopy versus computerized tomography. Table S2 The proportion of variation in the microbial taxonomy explained by individual factor based on permutational multivariate analysis of varianceof the Bray-Curtis dissimilarity matrix. Table S3 Microbial species associated with diverticulosis. Table S4 Sensitivity analysis of microbial species associated with diverticulosis, additionally adjusted for use of antibiotics and probiotics. Table S5 Microbial metabolic function associated with diverticulosis. Table S6 Sensitivity analysis for microbial metabolic function associated with diverticulosis, additionally adjusted for use of antibiotics and probiotics. Table S7 Microbial species significantly associated with asymptomatic diverticulosis according to anatomic location. Table S8 Microbial metabolic function significantly associated with asymptomatic diverticulosis according to anatomic location

## Data Availability

The metagenomic sequencing data have been deposited at Sequence Read Archive under BioProject accession: PRJNA784939. It can be accessed at https://www.ncbi.nlm.nih.gov/bioproject/PRJNA784939/ [59]. The individual-level metadata are not available due to reason of sensitivity and are available upon reasonable request from the corresponding author Dr. Hamed Khalili at hkhalili@mgh.harvard.edu. Researchers will be provided access to additional data within 6 weeks.
