# Supplementary material for: Gut microbiome structure and function in asymptomatic diverticulosis
Source: Genome Med. 2024 Aug 23;16:105. doi: 10.1186/s13073-024-01374-9 (PMC11342677; doi:10.1186/s13073-024-01374-9)
Supplement: Supplementary file 1 — Additional file 1: Supplementary results and Figs. S1–S4. Fig. S1 Microbial species and asymptomatic diverticulosis—sensitivity analysis. Fig. S2 A) Alpha diversitybetween asymptomatic diverticulosis at right colon, left colon, versus healthy controls; B) Principal coordinates analysisof study participants based on Bray-Curtis distances between gut metagenomic species profiles. Fig. S3 Taxonomic profiles of the gut microbiome significantly associated with asymptomatic diverticulosis according to anatomic sites. Fig. S4 Metabolic pathways and diverticulosis according to anatomic sites [file 13073_2024_1374_MOESM1_ESM.docx]

**Supplementary Materials**

**Gut microbial structure and function in asymptomatic diverticulosis**

**Authors**: Xinwei Hua^1,2,3^, Jessica McGoldrick^2,3^, Nour Nakrour^4^, Kyle Staller^2,3^, Daniel C Chung^2^, Ramnik J Xavier^,5 #^, Hamed Khalili ^2,3,5,6 #^

**Affiliation**:
^1^Department of Cardiology, Peking University Third Hospital, Beijing, China

^2^Division of Gastroenterology, Massachusetts General Hospital and Harvard Medical School, Boston, MA, USA

^3^Clinical and Translation Epidemiology Unit, Massachusetts General Hospital and Harvard Medical School, Boston, MA, USA

^4^Department of Radiology, Massachusetts General Hospital and Harvard Medical School, Boston, MA, USA

^5^Broad Institute of Massachusetts Institute of Technology and Harvard, Cambridge, MA, USA

^6^Institue of Environmental Medicine, Karolinska Institutet, Stockholm, Sweden

^#^Co-senior authors

**Correspondence**

Hamed Khalili, MD, MPH

Crohn's and Colitis Center, Massachusetts General Hospital

165 Cambridge Street, 9th Floor, Boston, MA 02114 United States

Institue of Environmental Medicine, Karolinska Institutet

C6 Institutet för miljömedicin, C6, CVD-NUT-EPI Wolk, 171 77, Stockholm, Sweden

Phone: 1- 617 726 7933

Fax: 1- 617 726 3080

Email: [hkhalili@mgh.harvard.edu](mailto:hkhalili@mgh.harvard.edu); [hamed.khalili@ki.se](mailto:hamed.khalili@ki.se)

**Supplementary results**

***Compositional differences in the gut microbiome between right- and left-diverticulosis and controls***

In per-feature analysis, although there was no significant difference of microbial species between left- vs right- sided diverticulosis, the relative abundance of five species were significantly different between left-sided diverticulosis and controls (*q* value <0.25, **Additional file 1, Figure S3A; Additional file 2, Table S7**). Among these, *P. copri* was enriched in controls (*q* value= 0.12) whereas several other species were more abundance in patients with left diverticulosis, including *Erysipelatoclostridium ramosum* (*q* value= 0.14) and *Parabacteroides johnsonii* (*q* value= 0.22), and two uncharacterized species *Clostridium sp CAG: 299* (*q* value= 0.10) and *Firmicutes bacterium CAG:424* (*q* value= 0.22)*.* In comparison, right-sided diverticulosis had significantly higher abundance of the microbial species *Bacteroides Salyersiae* (*q* value =0.21, **Additional file 1, Figure S3B; Additional file 2, Table S7**), an anaerobe shown to be positively associated with higher circulating CRP levels ^42^.

***Functional differences in the gut microbiome between right- and left-diverticulosis and controls***

For functional potential of the gut microbiome according to anatomic sites, we observed several metabolic pathways involved in glycolysis and carbohydrate metabolism remained enriched in patients with left-sided diverticulosis, including pathways involving degradation of D-galactose (PWY 6317, *q* value=0.04, and PWY 66-422, *q* value=0.06, **Additional file 1, Figure S4A** and **Additional file 2, Table S8)**. Several common housekeeping processes were enriched in controls, such as the biosynthesis of acid sugar 3-deoxy-α-D-manno-2-octulosonate, key component of bacterial LPS (PWY 1269, *q* value= 0.08, **Additional file 1, Figure S4A** and **Additional file 2, Table S8**). For right-sided diverticulosis, microbial functions involved in amino acid biosynthesis (PWY 5505, *q* value=0.08), carbohydrate metabolism (GLUCUROCAT PWY, *q* value=0.09; PWY 7242, *q v*alue =0.13; GALACT GLUCUROCAT PWY, *q* value=0.13), and vitamin B12 synthesis (COBALSYN PWY, *q* value=0.12, **Additional file 1, Figure S4B** and **Additional file 2, Table S8**) were enriched in right-sided diverticulosis as compared to controls.

**
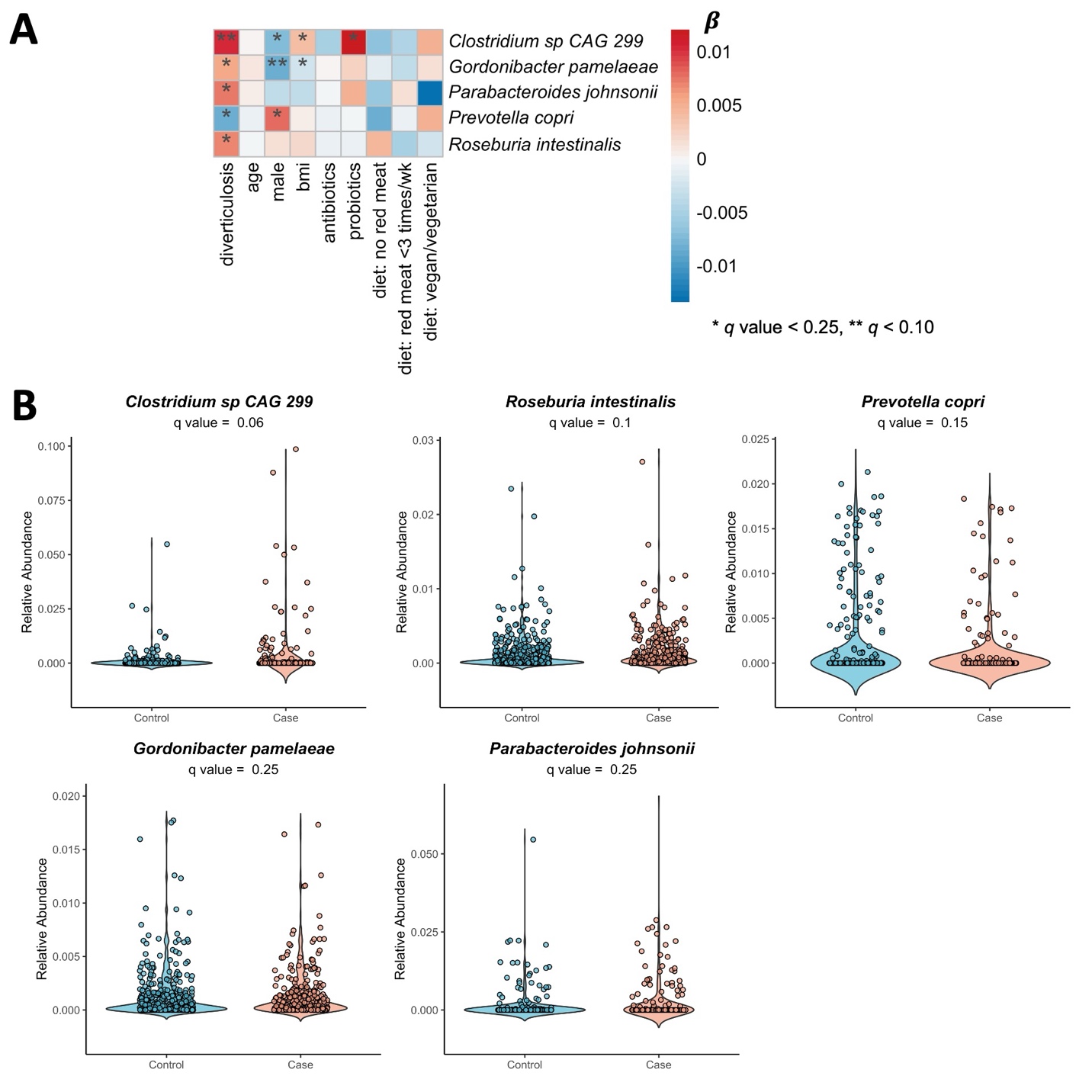
Supplementary Figures**

**Figure S1. Microbial species and asymptomatic diverticulosis – sensitivity analysis.
A.** Association between the relative abundance of fecal microbial species and asymptomatic diverticular diseases adjusted for age at colonoscopy, sex, body mass index (BMI), dietary patterns (limited red meat, no read meat, or vegetarian/vegan vs standard diet) and use of probiotics in the past two months and use of antibiotics in the last year using the multivariable linear mixed model. **B.** Relative abundances of microbial species significantly associated with asymptomatic diverticulosis (*q* value<0.25).

**Figure S2.** **A)** Alpha diversity (Chao1 index) between asymptomatic diverticulosis at right colon, left colon, versus healthy controls; **B).** Principal coordinates analysis (PCoA) of study participants based on Bray-Curtis distances between gut metagenomic species profiles.

**Figure S3. Taxonomic profiles of the gut microbiome significantly associated with asymptomatic diverticulosis according to anatomic sites.** **A**) Microbial species significantly different in relative abundance between left asymptomatic diverticulosis (n=201) and controls (n=400). **B**) Significant association between microbial species and right asymptomatic diverticulosis (n=73) vs controls (n=400).

**Figure S4.** **Metabolic pathways and diverticulosis according to anatomic sites.** Metabolic functions of gut microbiome significantly different between **A)** left-sided diverticulosis vs controls; **B)** right-sided diverticulosis vs controls (q values <0.25).
